# What are analog bulletin boards used for today? Analysing media uses, intermediality and technology affordances in Swedish bulletin board messages using a citizen science approach

**DOI:** 10.1371/journal.pone.0202077

**Published:** 2018-08-27

**Authors:** Christopher Kullenberg, Frauke Rohden, Anders Björkvall, Fredrik Brounéus, Anders Avellan-Hultman, Johan Järlehed, Sara Van Meerbergen, Andreas Nord, Helle Lykke Nielsen, Tove Rosendal, Lotta Tomasson, Gustav Westberg

**Affiliations:** 1 Department of Philosophy, Linguistics and Theory of Science, University of Gothenburg, Gothenburg, Sweden; 2 School of Humanities, Education and Social Sciences, Humanities Division, Örebro University, Örebro, Sweden; 3 VA (Public & Science), Stockholm, Sweden; 4 Independent researcher, Stockholm, Sweden; 5 Department of Languages and Literatures, University of Gothenburg, Gothenburg, Sweden; 6 Department of Slavic and Baltic Studies, Finnish, Dutch and German, Stockholm University, Stockholm, Sweden; 7 Department of Swedish, University of Gothenburg, Gothenburg, Sweden; 8 Department of History, University of Southern Denmark, Odense, Denmark; 9 Department of Swedish Language and Multilingualism, Stockholm University, Stockholm, Sweden; Bar-Ilan University, ISRAEL

## Abstract

Analog bulletin boards are omnipresent in Swedish urban areas, yet little systematic knowledge about this communication medium exists. In the shadow of the rapid emergence of digital media the analog bulletin board has received less attention than its digital successors, many of them having incorporated similar functionality with novel technical solutions. In this study we used a citizen science method to collect 1167 messages from bulletin boards around Sweden aided by school children and teachers, with the purpose of shedding new light on what is communicated on the boards, by whom, using what types of technologies and in what way the messages refer to other media. Results show that the most common messages are invitations to events, such as concerts, lectures and sports events, followed by buy-and-sell ads for goods and services. The most frequent sender is an association, for example NGOs, sports associations or religious communities. Almost half of the sampled messages were professionally printed, about forty per cent were made by home printers. Only six per cent of the messages were handwritten, almost exclusively by private persons as senders. Moreover, we show how the analog bulletin board has adapted to recent changes in media technology—a media landscape which is saturated with electronic- and mobile media. Further, the bulletin board still holds a firm place in a media ecology where local communication is in demand, and exists in parallel with electronic media. Close to forty percent of the messages contained hyperlinks to web pages and we found (and removed for anonymization purposes) more than six hundred phone numbers from the dataset.

## Introduction

Bulletin boards are ubiquitous to many modern urban areas and can be found throughout the world. The emergence of commercial advertising columns in Paris (Colonne Morris) and Berlin (Litfaßsäule) in the mid 19th century predate the first recorded patent for a “cork board”, which was approved to George Brooks in 1924 [1]. The diffusion of this innovation opened up the use of bulletin boards to a broader public throughout the 20th century, and inventions similar to the cork board are now used widely as a local communication medium.

In the age of electronic media, the interest in analog bulletin board has partly been overshadowed by the Bulletin Board System (BBS), a pioneering service in computer networking, which used the analog bulletin board as a model for facilitating many-to-many communications. The first dialup bulletin board was created in 1978 [2] in Chicago and offered similar functionality to those of its analog predecessor. With the rapid propagation of the World Wide Web in the mid 1990s, the BBS quickly declined in popularity, and today online services such as social media have integrated, modified and further developed the functionality of the analog bulletin board in new electronic media.

With the continuous growth and popularity of the internet, the analog bulletin boards might seem like an increasingly obsolete medium, one which is about to disappear and be replaced by digital solutions. However, despite the competition from electronic media, many public places still accommodate physical bulletin boards, which can be found in the streets, in libraries and schools, in private buildings, outside supermarkets, in cafés and bars. The bulletin boards are still in use but have not been widely researched, at least not in comparison with their electronic counterparts.

There exists a small amount of previous research centered exclusively on bulletin boards, which has created knowledge about the use of the medium with the purpose of developing recommendations for designing digital notice boards or displays [3–5]. All of these studies have had the board as such as the unit of analysis rather than the individual messages. Alt et al. studied what they call “Public Notice Areas” by taking photographs of 29 bulletin boards in Germany and Switzerland over a period of four weeks and by interviewing the owners of the boards [3]. Moreover, Taylor and Cheverst used a mixed method of surveying the bulletin boards in a rural village in North West England in addition to having “discussions with residents” about their use, and what implications such use had for design patterns [4]. In contrast to Alt et al., who advance the idea that analog bulletin boards are being progressively replaced by digital media, Taylor and Cheverst instead emphasize the many benefits of analog boards, such as “the obvious ease of use presented by paper flyers, the affordability of large displays, and their durability in a public or outdoor environment”, as well as the greater flexibility in placing messages [4]. Furthermore, Churchill, Nelson and Denoue used a similar method of talking to local inhabitants about their use of “community bulletin boards” in the San Francisco area, both in public places and in workplaces, in combination with observing the boards in their locations. Their results show that the boards were primarily used for communication that was locally relevant, and were closely integrated with community life [5].

In addition to the above-mentioned studies, within the field of linguistic landscape research, which examines the display of written texts in public spaces, many studies include notices and messages of the kind posted on bulletin boards in the sample data. Mostly, this is made as a means to contrast what is seen as “bottom-up” language usage as produced by private individuals and small associations with “top-down” language usage, as expressed in institutional and large-scale commercial signage. So far, however, only one study has dealt exclusively with notice boards. By documenting the content and changes of two “community” notice boards over one year, Peck and Banda [6] discuss patterns of “self-marketisation” and place-making in the post-apartheid neighbourhood of Observatory in Cape Town, South Africa. Their results show how new immigrant inhabitants in the neighbourhood deploy non-normative writing and orthography for upward social mobility; the “‘orthographic errors’ actually index the foreignness desired by middle-class residents in Observatory”[6].

As shown by these studies of linguistic landscapes, to understand in depth what is actually posted on the bulletin boards, each message has to be individually classified and transcribed[3–5]However, in order to be able to say something on the bulletin board as a medium we needed a large sample of data and relied on the help of citizen scientists in the shape of school pupils. Our study thereby contributes to the emerging field of citizen science that examines public language use (cf. Purschke [7,8], and Svendsen [9]). In this article we will present empirical data that aims to shed new light on an old medium in a contemporary media ecology, one which is saturated with mobile and digital media technologies. In contrast to most previous studies of bulletin boards, we do not have a design perspective in mind, but rather we aim to create new knowledge about the contemporary use and context of analog bulletin boards in Sweden as they occur in their cultural setting, and how they relate to other media. Moreover, though our primary concern in this article was with the use and content of the boards, the project as a whole was very much concerned with “facilitating democratic participation and increased public understanding of science” [9].

## Objectives

The purpose of this article is to inquire into the contemporary use of analog bulletin boards in public spaces in Sweden. The following research questions are posed:

RQ 1: Who is the sender of bulletin board messages and what is communicated?
RQ 2: What are the material properties of bulletin board messages and what affordances and constraints do these entail?
RQ 3: How do the analog bulletin board messages connect to other media (intermediality) and what forms of inventions emerge as attempts to expand the medium and to overcome its constraints?

The research questions will be answered with 1167 valid observations collected by citizen scientists consisting of school children in Sweden. The dataset is attached to this article as open data along with all source code. The paper also contains a discussion contributing to the above mentioned previous research [7–9] on the obstacles as well as the benefits of utilizing a citizen science methodology for collecting and classifying data in the social sciences and humanities.

### Affordances and constraints of the analog bulletin board

On a societal level, the analog bulletin board can be understood as a form of mediated “publicness” [9], where local messages are posted in a public sphere. However, unlike books, newspapers and broadcast media (television, radio), the bulletin board is made up of short messages posted by almost anyone with basic literacy skills. This way, it bears resemblances with contemporary online social media. Or rather, today’s social media have incorporated many of the features of the older analog bulletin boards. In a similar way as the Graphical User Interface of a personal computer borrows from analog media (files, folders, trash bins et. cetera), there are inherited features of the analog bulletin board in many electronic and networked media services that became popular with the adoption of the internet at the turn of the millennium.

The analog bulletin board has a variety of *affordances* [10–12] that make communication possible. Firstly, the boards are public communication technologies that are virtually open to anyone in the sense that they require no prior permission or sign-up for posting or reading messages. Messages can be produced at a very low cost, and distributed with little effort, at least in the local area. Secondly, what can be regarded both as a restriction (see below) and a potential is the geographical position of the board. Its placement inherently targets a local audience that is particularly desired in for example buy- and sell ads, lost- and found messages and invitations to local events. Electronic media often attempt to emulate this affordance, for example by generating “events near you” on Facebook or local categories on Craigslist and Ebay. Another affordance of bulletin boards is their large degree of freedom with regards to the form and content of the messages. Jones notes that bulletin boards have multimodal affordances, especially because they serve both as a place where you can post content, and as a site of interaction between the readers [13]. There is no prior censorship or requirement concerning language, use of images, drawings or typesetting, nor are there formal rules on length or topic of the messages.

However, as Norman has argued, affordances also are limited by both physical and cultural constraints [12]. Physical constraints of bulletin boards include:

- To publish or view messages, both the sender and receiver must be in physical proximity of the board.
- The board requires additional technologies of production, such as pencils, home or professional printers, and paper sheets, as well as adhesive technologies to attach the message to the board, such as sticky tape, staples or pins.
- Messages cannot be copied by the medium itself which limits their reach. Instead, additional technologies must be used, for example using personal notebooks to manually copy contact information, or more common today, using the camera of a smartphone to copy the entire message electronically.
- Although it targets anyone as the sender or viewer of messages, the bulletin board is essentially a one-way (mass-)communication medium. To afford a communicative feedback mechanism, each message needs to utilize peripheral media (intermediality), such as telephones, addresses, e-mail or websites.
- The bulletin board does not record any form of meta-data with regards to the audience’s consumption of the messages. Thus, it is not possible to measure the effectiveness of individual messages (except for tear-off strips, see below).
- Messages cannot be updated or changed easily (the sender has to revisit the board). This implies that information can be outdated or that some viewers might miss newer information added later on.

In the usage of bulletin boards, there are also a number of cultural constraints that are less rigorous, yet play an important role in the practical use of bulletin boards:

- In general, messages should be short and limited to a single piece of paper.
- Boards have different implicit or explicit conventions for the duration and removal of a message. If a board is full, the user has a degree of freedom in choosing whether to remove other messages or post a new message on top of an existing message. Often the owner of the board will perform regular cleanups by removing old messages. In some cases there might be a restrictive selection by the owner of the board with regards to which types of messages are allowed.
- Bulletin boards have developed a number of genres that messages often adhere to, such as job-seeking notices, buy- and sell ads, invitations, lost- and found notes, etc. While these genres are not absolute constraints, they influence which types of messages are perceived as suitable or possible to post on a bulletin board.
- Not only the owner of the board, but virtually any receiver of the messages posted, may decide to remove a message after it has been posted. The freedom of posting thus also comes with the drawback of a “freedom of removal”.

## Method

In this study we used a citizen science approach to collect and classify 1167 bulletin board messages in Sweden during the fall of 2016. This allowed us to reach a much wider territory for data collection and to collect a much larger number of observations. From the point of view of published articles, citizen science as a method has not been widely adopted in the humanities and social sciences [14]. However, several projects have used forms of crowdsourcing for humanities research that could be included in the concept of citizen science. For example, projects such as Art Detective aim to include citizens as sources of expert knowledge (niche-sourcing, e.g. on art https://www.artuk.org/artdetective/). Other projects encourage citizens to contribute to collections with their own materials (e.g. on history in the Bracero Archive, http://braceroarchive.org/). Another common model is to invite volunteers to work with a given dataset, with the tasks of transcribing, tagging, classifying or correcting existing data (e.g. transcription of historical texts in the Transcribe Bentham project, http://www.transcribe-bentham.da.ulcc.ac.uk/ or tagging images for archaeology in Global Explorer, https://www.globalxplorer.org/). A few examples of crowdsourcing games for research can also be found (e.g. http://www.metadatagames.org/). Like citizen science projects, citizen humanities projects can be hosted and developed individually or on common platforms such as Zooniverse (http://zooniverse.org). Svendsen [9] discusses the differences between crowdsourcing and what she calls citizen sociolinguistics: “Citizen sociolinguistics is not *per se* about crowdsourcing (meta)linguistic data”, but rather about “the engagement of nonprofessionals in *doing* sociolinguistic research”. However, Lingscape is also a crowdsourced app and project[8].

As Pocock et al. [15] and Cooper et al. [16] have shown, citizen science has become a widely used methodology in conservation and biodiversity research, and the results of these projects have often been used outside academic settings [17,18]. In this article we have used a method similar to studies that rely on recording species occurrences and distributions in biodiversity citizen science, but instead we use it for gathering bulletin board messages using a smartphone app. The comparison of course ends here, since we are working with cultural artefacts that are the product of human-technological interactions, which neither have a clear taxonomy nor exist outside the imprint of human culture.

### Inviting participating schools and sampling bulletin boards

**Fig 1:**
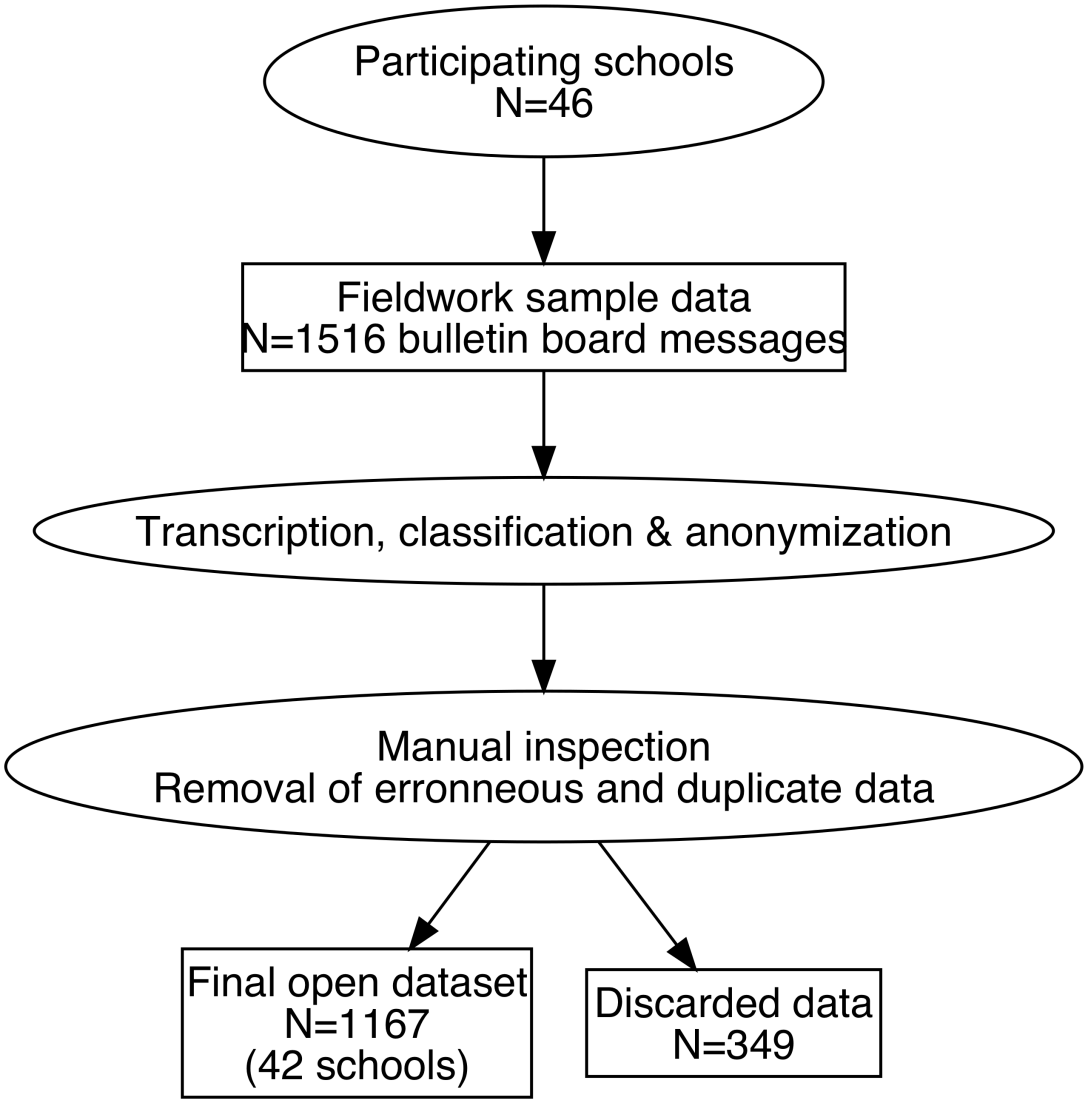
Data collection flow chart. Data collection, classification and verification. The valid data, with regards to raw images, were then inspected manually. All transcribed text was checked by a proofreader who ensured that the transcribed text corresponded to the actual image, preserving capitalization, punctuation and errors related to spelling and grammar. Only Latin script was transcribed, other scripts (Arabic, Farsi, Dari, Tigrinya, Thai etc.) were classified but not transcribed. Moreover, the data was classified by two researchers (see codebook in S2 Appendix), who worked closely together in order to resolve inter-coder reliability issues.

We made an open call to school classes to participate in the collection of data. 46 schools accepted the invitation and were sent general information about the research project along with background information, in order to facilitate classroom discussions about bulletin boards. The 46 schools were composed of 96 classes, of which 14 were grade 1–3, 48 were grade 4–6, 12 were grade 7–9 and 15 were grade 10–12 (high school). Additionally there were 7 mixed classes that spanned over several grades. We also sent each teacher detailed instructions on how to collect bulletin board messages using a mobile application. A web-based version of the mobile app, which is close to identical in layout, can be found at https://forskarfredag.se/massexperiment/anslagstavlan/. The instructions were written in a “teacher’s guide”, a small booklet with step-by-step instructions on how to download and install the mobile application, report a bulletin board message and how to ask questions. When teachers created accounts in the mobile app, they were informed of the purpose of the data collection, consented to the data being used for research, and were asked to provide contact information. Moreover, they agreed to the End User License Agreement of the mobile app, which states that the data collected will be used for research. The pupils themselves did not register for individual accounts, thus no personal data was collected, except the teacher’s e-mail addresses. Consequently, each school class only used one single account for logging into the app. We also set up a closed Facebook group to answer questions from the teachers and we also provided email support, neither of them frequently used. Data was collected during the period from 2016-09-15 to 2016-10-30. After removal of erroneous data (Fig 1), 42 schools had contributed with valid observations.

Most teachers reported that they integrated the citizen science project in the curriculum as a way of learning about communication, media and multilingualism in society. The preferred transportation for collecting data varied from using a bicycle to reach boards that were distant from the school to simply walking in proximity inside and outside the school building. Often the pupils worked in small groups, meaning that behind the camera there were several pupils collaborating. Since they all used the same login identity, it is not possible for us to know how many physical devices were used, a trade-off in data resolution that had to be made for privacy concerns. Many teachers mentioned that there was a lack of time to complete the task, since the scheduled time was too short to make the desired contribution. Three teachers who had initially agreed to participate, dropped out from the project due to time constraints. One teacher reported that they had aborted the collection because the pupils did not want to continue.

### Transcription, classification and verification of data

The raw data contained 1516 images of bulletin boards (Fig 1). We removed images that were taken of entire boards instead of a particular message, and we also removed empty or distorted images.

We also marked duplicate entries in the dataset, distinguishing between two kinds of duplicates. Firstly, there are “virtual” duplicates that are the results of two or more participants taking pictures of the same physical bulletin board message. These were removed from the dataset, since they were merely the result of a methodological error. However, there were also what we could call “physical” duplicates. These are the result of copies of the same message having been posted on several bulletin boards. These physical duplicates are interesting from the perspective of media technologies, since they indicate some sort of copying device (professional or home printer, photocopier etc.) and a form of “mass communication” where bulletin boards are used to reach wider audiences. We marked these physical duplicates by enumerating each duplicate (1, 2, 3. etc.). This way they can be either included or excluded from the dataset, depending on whether duplicates are relevant to the research question at hand. We also removed a small number of completely erroneous images where the school children had taken “selfies” and “funny pictures” instead of following the instructions.

In total, we collected 1167 valid observations, of which 130 were physical duplicates that occur more than one time in the dataset. The entire dataset is written as a comma separated values (CSV) file in S1 Appendix and can be analysed with a wide range of software, or read into the Python 3 Jupyter notebook in S2 Appendix in order to replicate the figures in this article or to make use of some pre-written search and visualisation functions.

### Representativity

The problem of the citizen science approach for collecting data is sometimes referred to as “sampling effort variation” [19], where volunteers are gathering information of value to researchers based on their respective effort capacity. In our case, our data points have a geographic reach that only stretches a few kilometers away from each participating school. As pupils, with a few exceptions, were instructed to perform the task of photographing bulletin boards during class hours, they were limited to reaching only boards within a short walking-distance from the school facilities.

Inviting schools to participate targeted each primary- and secondary school in Sweden; we sent one invitation letter to each school in May 2016. The addresses were obtained from the Swedish National Agency for Education. Furthermore, we sent personal invitations to teachers that had already participated in previous citizen science projects. Finally, we wrote press releases aimed at national print- and broadcasting media outlets that we were inviting participants for the project. These press releases were reported in a handful of local radio broadcasts and in some specialized magazines. This resulted in a self-selected sample, with 46 participating schools across Sweden.

**Fig 2:**
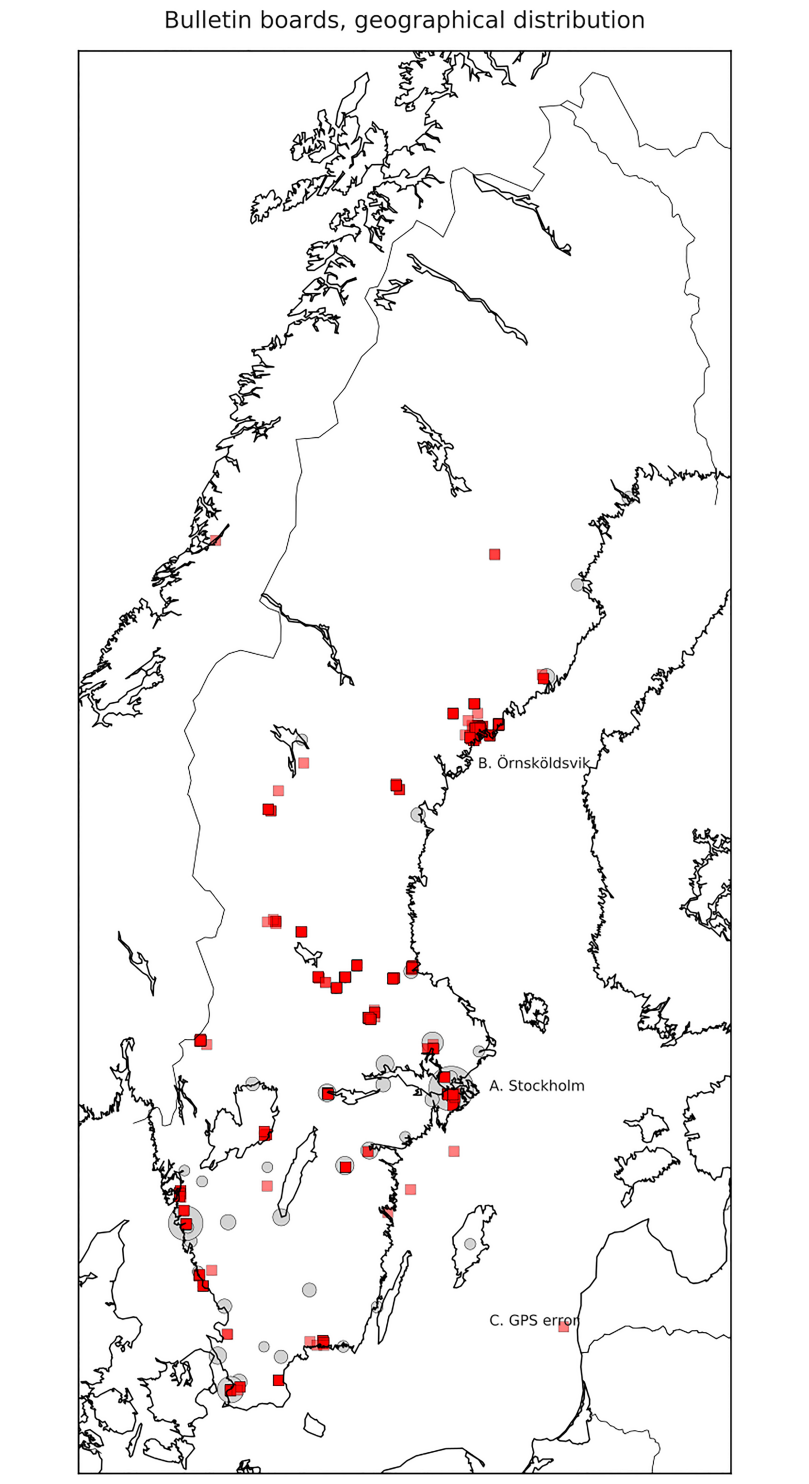
Geographical distribution of collected bulletin board messages (N = 1167). Each bulletin board message is plotted as a red square and cities with more than 50,000 inhabitants are plotted with grey circles.

However, the level of participation varied between schools. Partly this was an effect of how much time each teacher devoted to the task, but also some schools engaged more pupils in several classes, thus increasing the number of participating pupils. This ranges from only a handful of collected data points for the least active school to hundreds of observations made by highly engaged schools. As shown in Fig 2, we have plotted each bulletin board message on a map of Sweden. From this distribution of data points we can draw the following conclusions:

Large urban areas, such as Stockholm, Gothenburg and Malmö, are under-represented in terms of population demographics (see point A in Fig 2).

The city of Örnsköldsvik is highly over-represented (343 collected messages, point B in Fig 2) due to the fact that several school classes with a high level of engagement collected data, and all participants were high school pupils (age 16–20).

The location data is sometimes unreliable due to GPS performance of the devices used. As also shown in Fig 2 at point C, there are some observations appearing to be positioned in the Baltic Sea, thus must be regarded as noise.

Even though the data is not representative in correlation with demographics, the geographical distribution has a high reach. There are reported data points from north to south, coast and inland, in less populated rural areas as well as larger cities. Similar circumstances prevail for the Norwegian citizen science project on linguistic diversity where, according to Svendsen [9] the “total of 4500 pupils is clearly not representative for Norway as a whole, it is nonetheless a sizeable body and there is no obvious bias in who volunteered as citizen scientists and as respondents.” The latter can be questioned. In his discussion of the citizen science tool Lingscape, Purschke [7,8] distinguishes between the representativity of the data and the representativity of the population who volunteers as citizen researchers: “While the collected data itself is representative, it comes from a non-representative population, which introduces a bias (presumbaly a younger audience that might photograph different signs than older people) to the sample.” Importantly however, such bias can be countered by “collecting many different individual perspectivations of the landscape without imposing a specific research interest on our participants” (ibid.). That is, …

### Anonymization of data

All bulletin board messages collected were posted in public or semi-public spaces. However, the people posting messages could not anticipate that their messages would be digitized and transcribed. Moreover, bulletin board messages constitute what Klaus Bruhn Jensen calls "found data" [20], which means that the data is naturally occurring without the intervention of a researcher (as contrasted to "made data", for example surveys, interviews etc. that are initiated by researchers). Such found data need to be treated with special care because there is no prior informed consent procedure.

Therefore, all personal data was removed during the transcription phase. With regards to the bulletin board messages such information included:

- Telephone numbers, email addresses, private street addresses, personal social media usernames.
- Vehicle registration plates (often stated in buy-and-sell ads).
- Given names (with the exception of pets and public figures, such as artists, performers, etc.).

### Data and code

The final anonymized and verified dataset was written to a csv-file (S1 Appendix) that could be processed with the Python 3 programming language. All code was executed and documented as a Jupyter Notebook to ensure the replicability of the analysis (S2 Appendix). Both the source data and the computer code are released as open data and open software.

## Results

### RQ 1. What is communicated by whom?

The bulletin boards are used primarily by various associations (43.8%), companies (28.4%) and private persons (14.1%), and to a lesser extent by authorities, municipalities and regional services. Many of the companies found in the messages are small, privately or family owned companies. 5 of the messages mention the so-called F-tax certificate (f-skattesedel) that is used by self-employed entrepreneurs in Sweden.

**Fig 3:**
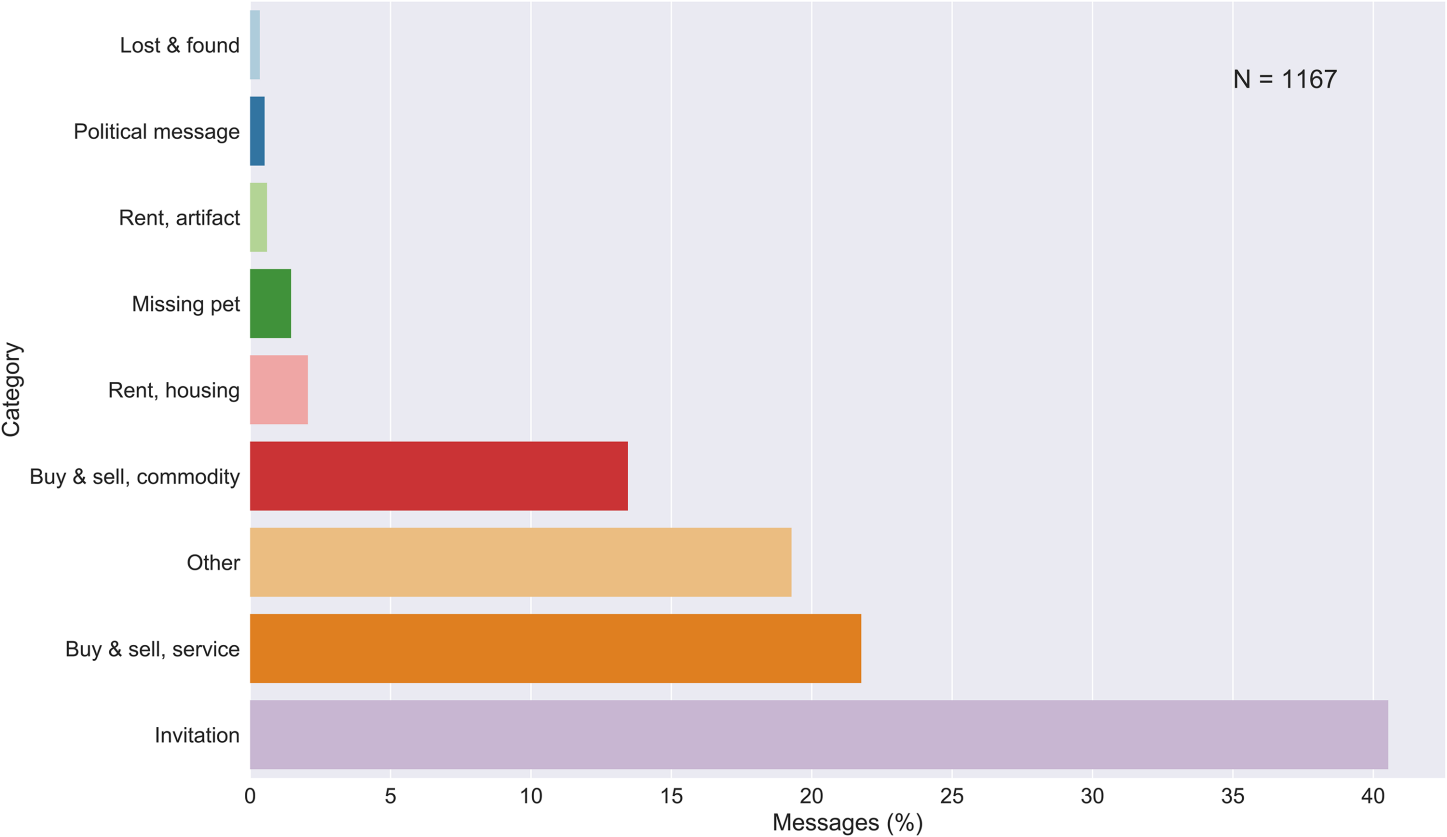
Message categories (N = 1167). Includes duplicate messages (see section “Transcription, classification and verification of data”). The category “Invitation” is composed of several sub-categories (such as “Invitation sports event”), see S2 Appendix.

The most common category of message (Fig 3) is an invitation (40.5%, several forms of invitations merged together, see S2 Appendix). However, if buy- and sell ads are combined for both commodities (13.5%) and services (21.8%), this category is almost as large. This group is followed by a big group of messages categorized as other (19.3%), with the remaining groups of houses and flats for rent, missing pets, rental of artifacts, political messages, and lost- and found messages occurring much less frequently.

The types of messages are not distributed equally among the different senders. While invitations mostly stem from associations and companies, messages about lost-and-found artifacts or pets are almost exclusively written by private persons. Almost all of the messages for renting living spaces are also contributed by private persons, whereas artifacts are more often rented out by companies. A similar difference occurs in the buy-and-sell messages: many private messages about commodities can be found, whereas services are often offered by companies. Many of the messages by authorities, municipalities, region, state, or others were classified as “other” content.

The two biggest groups of contributors, associations and companies, contribute to many different categories of messages. However, for associations, the largest contribution is invitations, whereas for companies, the largest contributions are in ads for buying and selling of services.

In terms of language, our sample showed Swedish language to be very dominant. Only 37 messages (3.2%) were written in another language, 28 of them in English, 4 in Norwegian, 3 in Finnish and 2 in Arabic. Moreover, 33 messages contained two languages, most commonly a mix of Swedish and English. 8 messages were classified as multi-lingual, containing more than two languages. Here we find examples of immigrant languages (Arabic, Dari, Somali) and typical tourist languages (English, German). As discussed in the Methods section, however, these results should be interpreted with caution, since our sample most likely underrepresents areas where languages other than Swedish are common.

In our material we find some organisations that are specific to Swedish cultural life, which combined make up a significant portion of the data. For example, the PRO (the Swedish National Pensioners’ Organisation) occurs on 17 unique messages. Hembygd- (as in Hembygdsförening and Hembygdsgård, an open air museum with local heritage focus) occurs 18 unique messages, Folkets Hus (community centers, originally affiliated with the labour movement) occurs in 10 unique messages. ABF (the Workers’ Educational Association) have posted 22 unique messages and Studieförbundet Vuxenskolan (study circles for adult education) account for 13 messages.

### RQ 2. Material properties and affordances

**Fig 4:**
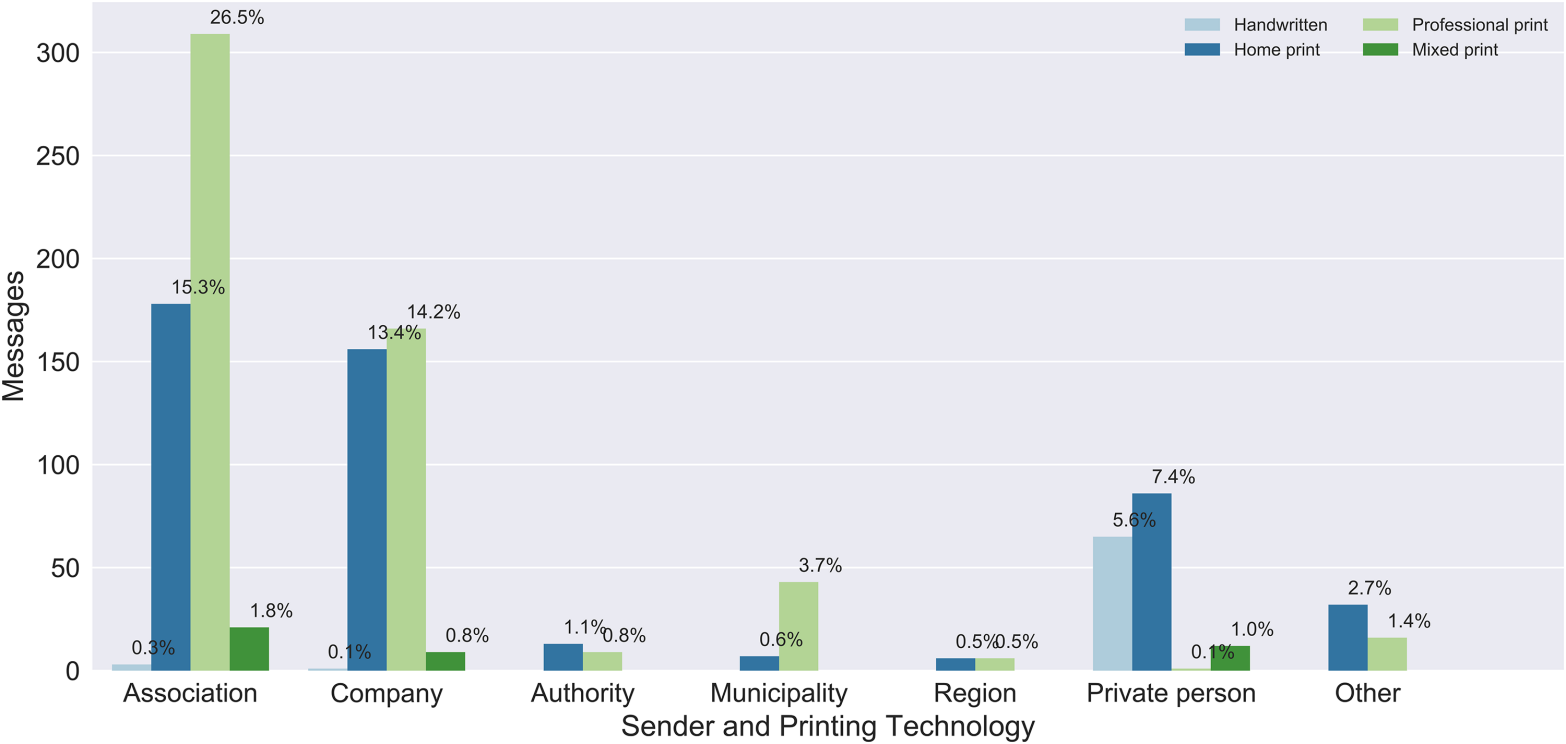
Sender and printing technology (N = 1167). Associations rely mostly on professional prints while companies use home (or office) printers to almost an equal extent. Handwritten messages can almost exclusively be found among private persons.

Fig 4 shows the printing technologies that different senders of messages use. Associations mainly use professional printing for their messages. More than a quarter of the messages found on bulletin boards (26.5%) are professionally printed posters with an association as the sender. Businesses, on the other hand, use professional prints and home printed messages to an almost equal extent. Since the companies using bulletin board messages are often small, they might be less prone to invest in professional prints than associations that might have larger budgets or access to printing services. The businesses found in the dataset include for example yoga-teachers, gyms, restaurants, rental services, or craftsmen. Associations comprised small local associations, groups, and clubs (e.g. sports groups, heritage centres, after school activities for children) as well as non-profit organizations (e.g. churches, libraries, relief organizations).

Handwritten messages are almost exclusively authored by private persons. However, such notes only comprise less than six per cent of the entire dataset, and private persons more commonly use home printers. Digital handwriting, or script fonts, are however frequently used in the digitally produced texts. It can serve as a resource for adding a personal, intimate, or authentic tone to the message, or for creating ideas of childishness, or play.

Overall, 42.3% of the dataset were classified as home prints and 47.8% were classified as professional prints. Almost 80% of the collected messages had been printed in color. A small amount of the messages (3.7%) used a mix of handwritten and printing technologies, for example manually adding details to an inkjet or professional print-out.

Most of the recorded messages contained some form of image, with 40.2% of the images containing photographs, 19% containing clipart and 12.2% containing combinations of different types of images. 3.4% of the messages contained hand-drawn images or illustrations, whereas 24.2% contained no illustration at all. Images included photographs directly related to the messages’ content (e.g. photographs of missing pets, portraits of performers, images of objects to be sold) but also more generic photographs used symbolically or to catch viewer’s attention.

As for adhesive technologies used to attach messages to bulletin boards, only a small proportion of the sampled messages had been attached using tape or other technologies such as magnets or clips. Most of the messages had been put up using push-pins (48.6%) or staples (31.4%), with a difference between indoor bulletin boards where pins were prevailing and outdoor boards where staples were more common. In comparison to Alt et al. [3] we did not find the type of “Scaffolded Classifieds Display”, which provide pre-printed messages and specially designated frames where the messages can be inserted. Sticky tape was quite rare (7.5%) as an adhesive technology. Also, we classified 6.1% as “other” in the dataset. These mostly consist of messages that were attached to locked boards (behind glass doors) using magnets.

The analog bulletin board is restricted to a confined geographical position. To cover a larger area, the messages have to be copied physically and then distributed to other boards. In our sample 130 of 1167 (11%) messages occurred more than once and were identified as physical copies of the same message in different locations. More than half of these copies were invitations, for example to concerts and events, and were posted by associations or companies as professional prints. Most of the duplicates were only found two times, but there were some messages that occurred up to six times on several boards. However, our methodology limits the certainty of such numbers, since the school classes only collected messages in proximity to their schools. Multiple copies of the same message would probably extend over a greater area as numbers increase, thus falling outside the reach of our volunteer contributors’ walking distance.

### RQ 3. Intermediality and enhancements

Despite cultural and physical constraints, bulletin boards still seem to be used frequently in Sweden. In the interplay between affordances and constraints, the analog bulletin board has given rise to some practices and enhancements that attempt to expand the use of the medium. Users of bulletin boards combine their messages with other media to expand their opportunities for communication (intermediality) and make use of inventions to combine the advantages of different technologies, for example adding tear-off notes with phone numbers or using pre-printed posters with handwritten details.

**Fig 5:**
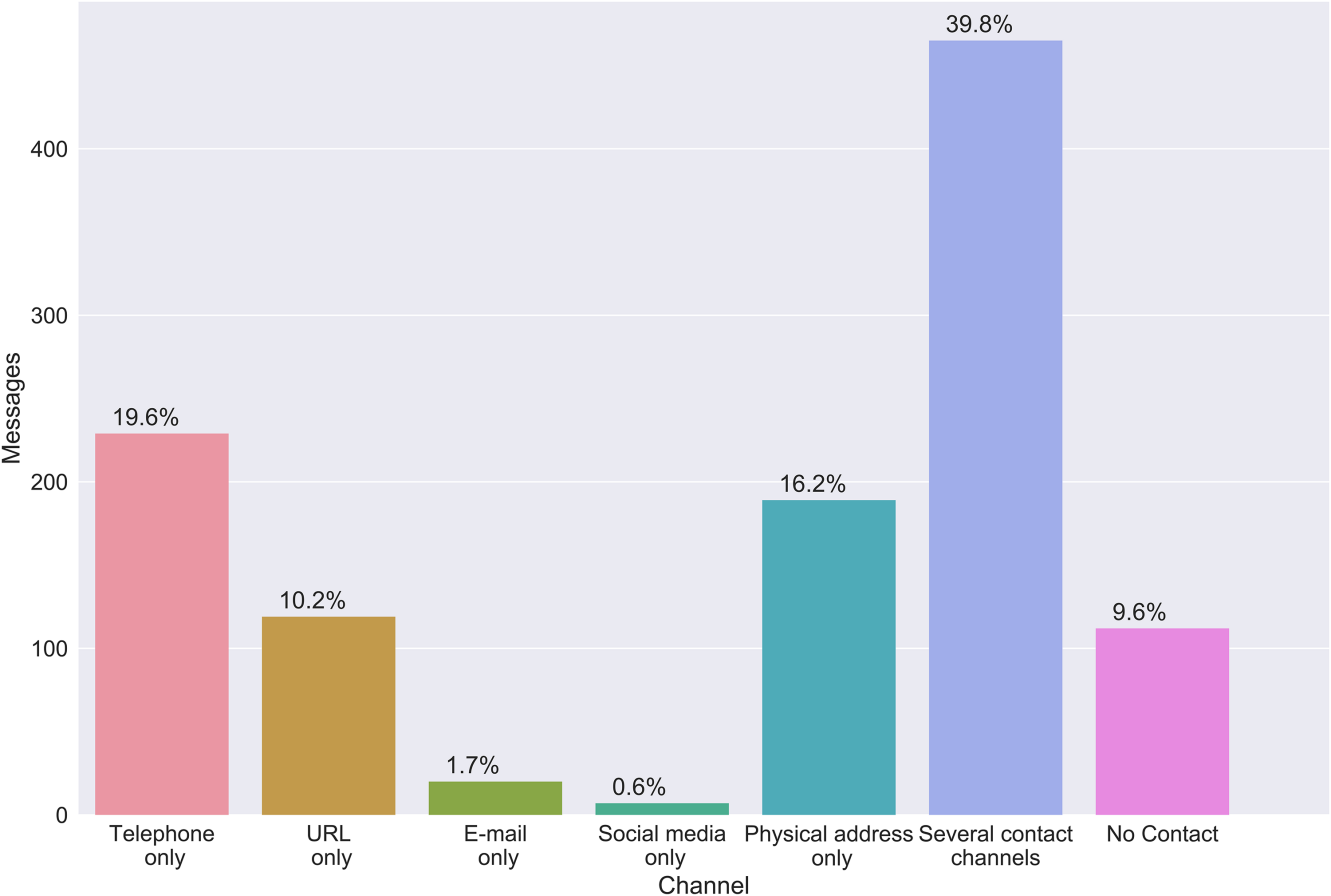
Intermediality on bulletin board messages (N = 1167).

With regards to intermediality, almost 39.8% of the messages have multiple contact channels to overcome the limitations of the physical constraints that the bulletin board has as a medium (Fig 5). These combinations of contact channels include phone numbers, email addresses, websites, social media accounts and physical addresses, which prompts the reader of the message to switch to another medium for feedback.

The most common practice is to use more than one medium to connect the messages, mostly for the reason of providing an auxiliary feedback mechanism that opens up a channel for two-way communication. Phone numbers and email addresses have this function. However, URLs are often used for similar purposes, either to supply more information or providing a feedback channel. Physical addresses are common when announcing events such as concerts or meetings.

As a single medium, the telephone (mobile and landline combined 19.6%) is the most common way of establishing a link between a board message and the sender, followed by the mention of a physical address (16.2%). 9.6% of the messages contained no contact information at all. Very few messages opted for e-mail or social media as the only contact information, but instead combined these auxiliary media with phone numbers and URLs.

In the overall dataset, Facebook was mentioned in 63 messages (5.4%). Moreover, we removed 629 phone numbers of private persons and 190 personal e-mail addresses from the dataset for anonymization purposes. Both phone and e-mail enable a feedback mechanism suitable for person-to-person communication, and are widely used in buy-and-sell ads and lost-and-found animals messages.

We also coded the presence of a URL separately. In total, 461 messages (39.5%) contained URLs, which indeed indicates a progressive intermedial integration between the analog bulletin board and the world wide web. With this particular affordance, not only is a potential feedback channel opened. But it also provides a means of overcoming the constraints in space of the physical message and enable additional multimedia experiences that are possible on the internet.

A special example of intermediality is the occurrence of QR-codes in some of the bulletin board messages. Although rare, some messages included QR-codes to connect viewers to other resources on the internet. What is noteworthy about QR-codes compared to a written phone number or URL, is that the immediate availability of a smartphone and internet connection is presumed. Viewers need to have access to mobile technology on-site to be able to follow the link presented in form of an QR-code, rather than choosing how to record and follow the link (on-site or later on). This might explain why only very few QR-codes were found in the dataset, and why they were not used exclusively but as an alternative way of accessing URLs.

**Fig 6:**
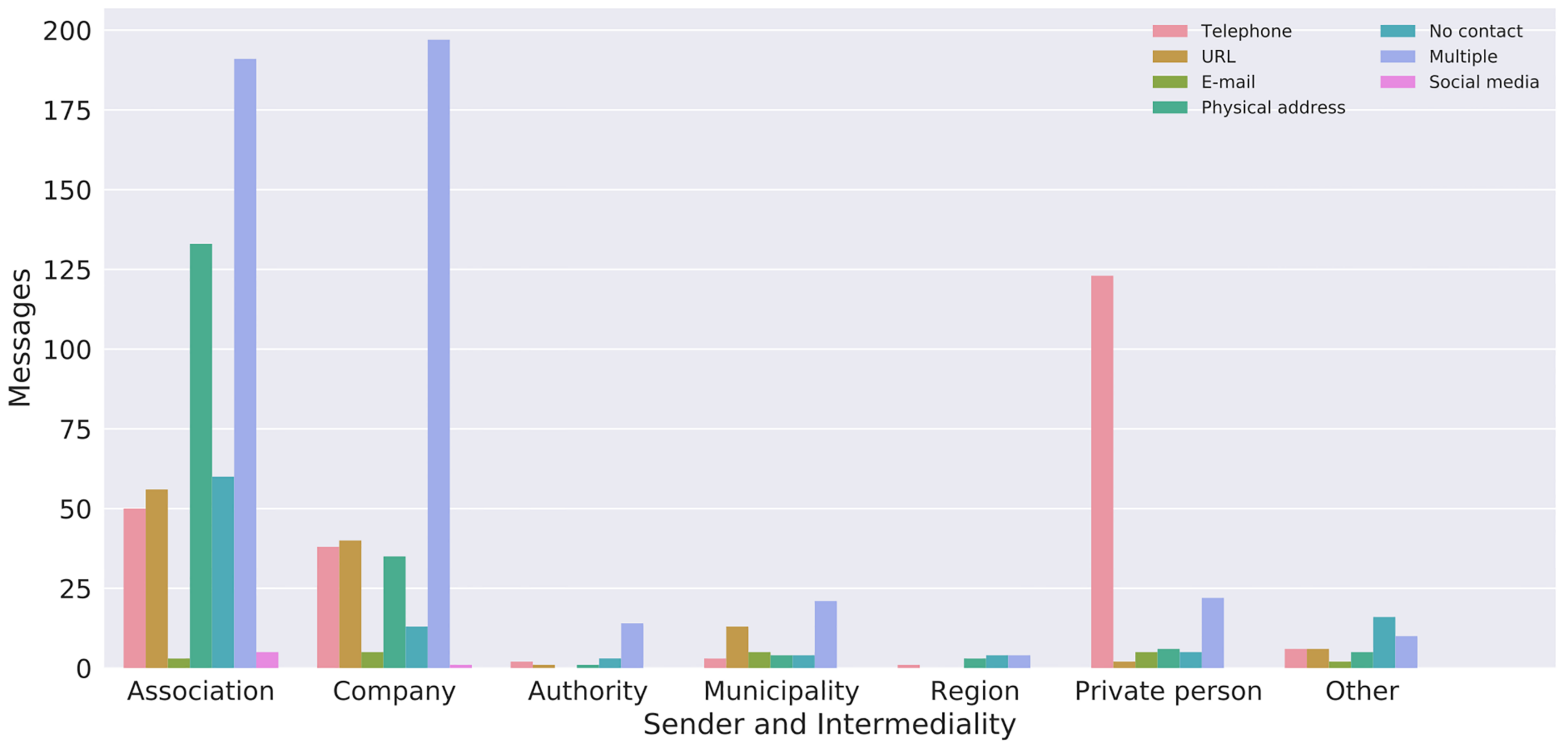
Intermediality and sender (N = 1167).

In cases where contact information is absent, this is often an effect of the hyper-local affordance of a bulletin board. Several messages in this category contain information that is only valid or comprehensible in its immediate local context. For example, a note informing that fishing is prohibited in a lake, a supermarket stating the rules for buying alcoholic beverages or a church announcing a concert—these messages concern the immediate location (lake, supermarket, church) and have thus implicitly already stated their location as a point of contact. Without knowing their physical location, these messages completely loose the context needed for comprehension. These hyper-local messages are most commonly sent by associations (Fig 6).

The practices of intermediality differ quite sharply depending on who is the sender of the message. As shown in Fig 6, private persons primarily use telephone alone as a way of establishing a link between the bulletin board message and the sender. However, the telephone must be understood as a medium with multiple affordances, as mobile phones can transmit and receive text messages in addition to phone calls. Increasingly, mobile devices are also used for monetary transactions by using bank transfer applications (which are widely used in Sweden) that can be used conveniently, especially for buy-and-sell ads where a successful communication leads to a purchase or sale of an item or service.

The intermediality of messages sent by associations and companies stand in stark contrast to the uses among private persons. Most commonly these messages include multiple feedback and contact channels, such as web URLs (more common for companies than for associations), e-mail, telephone and physical addresses. In the case of associations, we often find invitations to concerts, sports events or religious gatherings. Here the recipient of the message is invited to appear at a specific time and place and website URLs or e-mail addresses are only used for additional information or ticket purchases. The physical address as the only feedback mechanism is found primarily in messages by associations.

**Fig 7:**
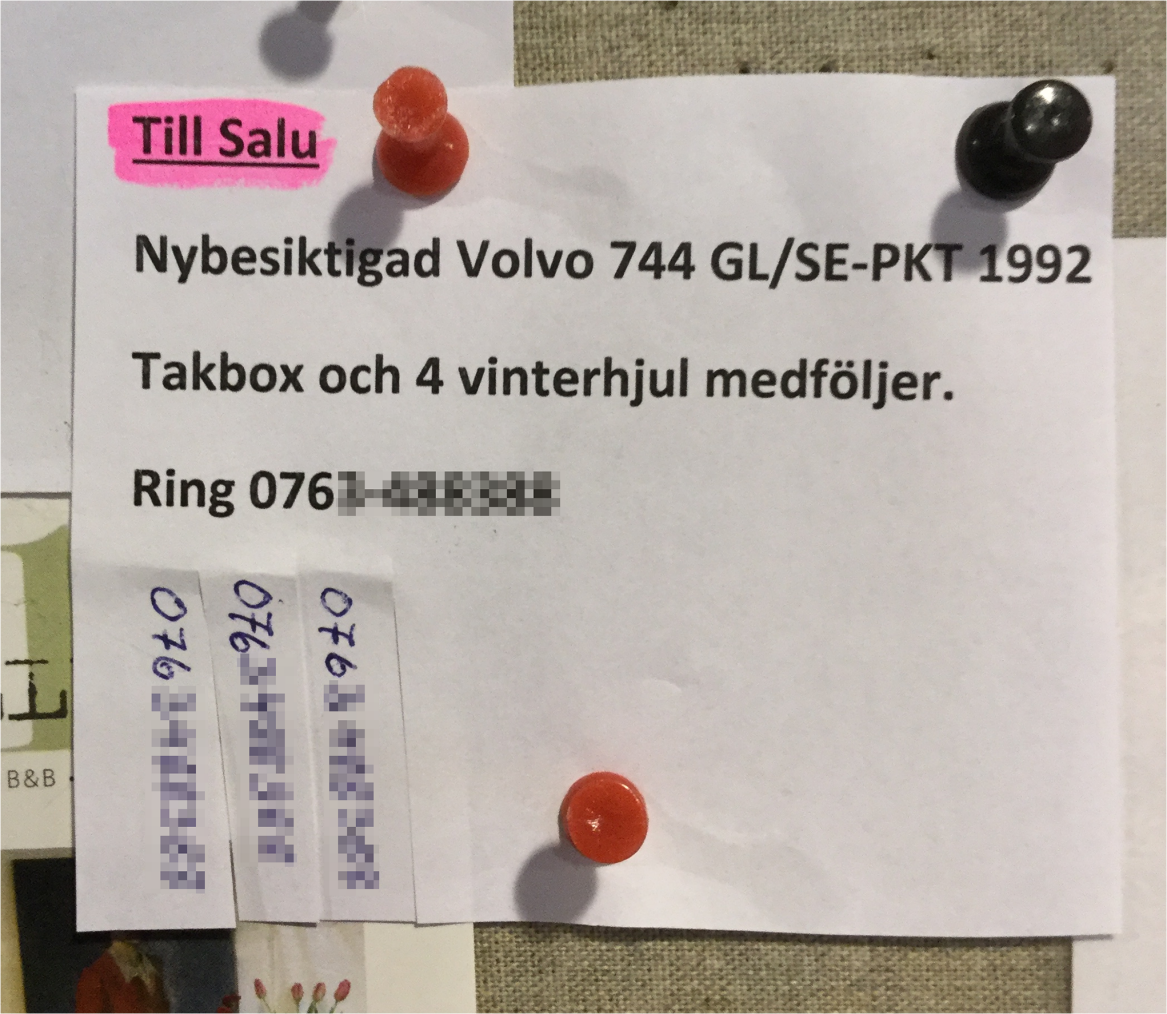
Tear off strips. Example of a buy and sell ad with vertical tear-off phone numbers. The message was posted on a supermarket’s notice board and reads “For sale. Recently inspected Volvo 744 GL/SE-PKT 1992. Roof box and 4 winter tyres included. Call 076X-XXXXXX”. The message both utilizes the tear-off convention and includes a horizontally printed phone number. It has been created using a home printer (which in turn requires additional hardware and software) but with highlighting and vertical phone numbers added manually (requiring pens as well as scissors to create the tear-off notes).

Bulletin boards make it easy to reach a large number of viewers but in order to receive feedback from viewers, other communication channels have to be added. One of the constraints in making other channels available is that viewers might not have the time or the necessary devices to record the contact information. A common way to overcome this limitation is the creation of tear-off strips (as previously observed in California by Churchill, Nelson and Denoue [5], and in Germany and Switzerland by Alt et al. [3], here referred to as “takeaway tabs”), often used for phone numbers but even possible for email addresses or URLs. The information is written repeatedly and the paper message modified with the aid of scissors to enable viewers to simple tear off and keep a small note containing the information for later use. Fig 7 shows a typical example of tear-off strips with phone numbers, which are frequently used in buy-and-sell ads, but are also found in other forms of invitations to local events. The tear-off function also adds a unique form of “meta-data” for each message. The sender of the message can go back to the board and inspect how many phone numbers have been torn off. This way, the sender can approximate how many “views with interest” that the message has been exposed to. Moreover, based on the number of tear-off notes that have been torn of, the viewers of the message get an idea of the popularity of the message. Senders sometimes also draw on this extended media-knowledge in order to make their notes more attractive, and hence tear a few notes away already when posting the message.

**Fig 8:**
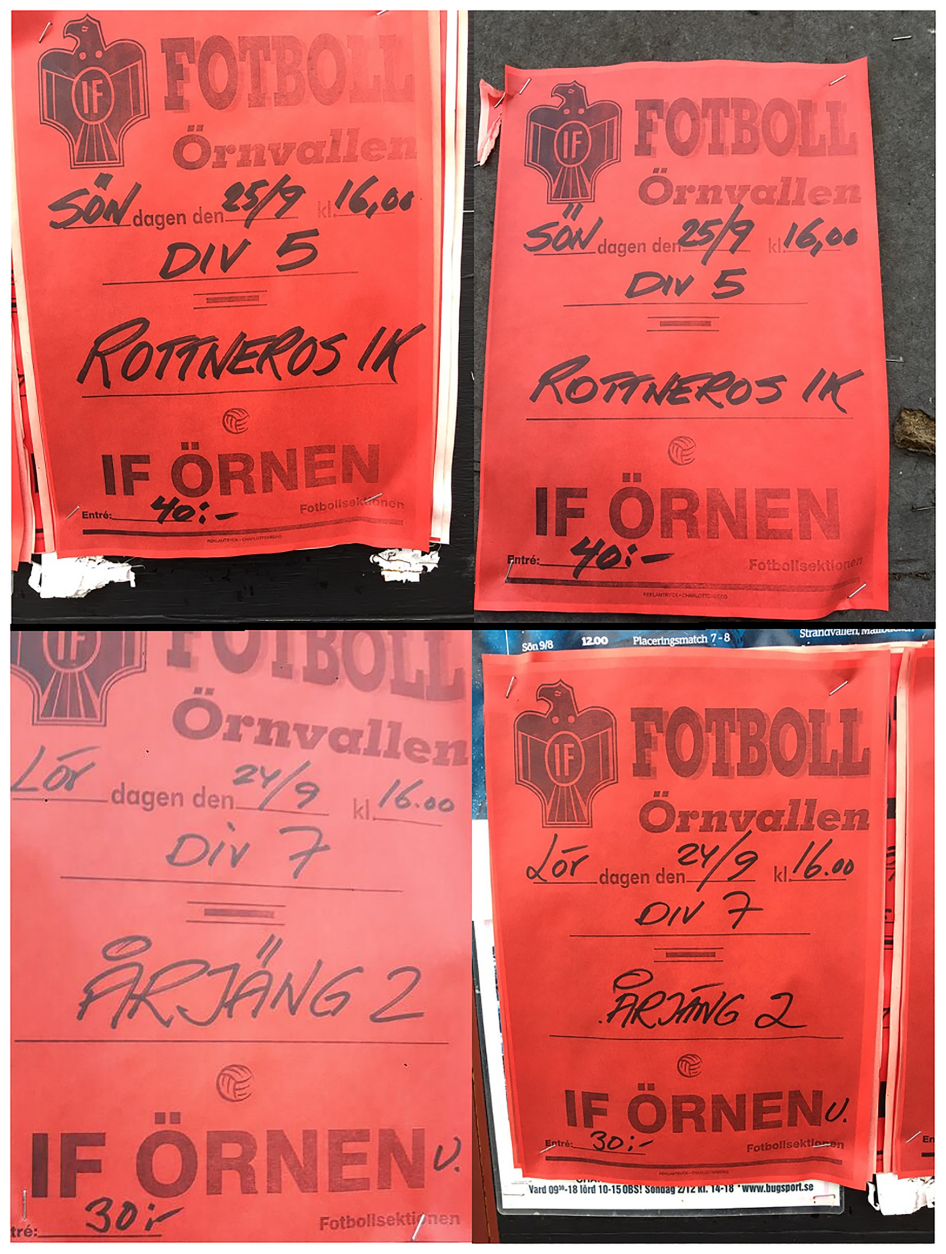
Multiplication affordances. Duplication of messages using a photocopier and a marker pen. Invitation to soccer games by the club “IF Örnen” occurring on Saturday and Sunday, with handwritten information about date, time, division and name of opposing team. The bulletin boards were recorded within a short walking distance of the soccer field.

43 bulletin board messages were classified as “mixed print”, using a mix of printed and handwritten information. These include mainly invitations and buy-sell ads. In some messages by private persons, handwritten information was used to add forgotten information later on, or to create tear-off notes (Fig 7). However, associations and businesses also use mixed printing to combine the advantages of professional printing with the flexibility and cheap/simple production of handwritten information. Examples of this include invitations to concerts or sports events where details like date or place where added to the poster by hand. Fig 8 shows an example of an invitation to a soccer match, a common type of message found in our dataset. Although the sender of these invitations (IF Örnen) also has an active Facebook page, the club still relies on the simple and cheap technology for creating invitations on local bulletin boards to target a local audience. As shown in Fig 8, there is a professionally printed original message (developed by a printing business called “Reklamtryck Charlottenberg”), which in turn is copied repeatedly using a photocopier (or similar technology). The co-occurrence of bulletin board messages and online messages indicate that the analog and digital media are used in parallel, but partly for different purposes. The Facebook page of IF Örnen contains a wide variety of additional information, such as match results, photographs and comments. However, the Facebook page has not been perceived as a complete replacement for the analog bulletin board, which is still in active use.

## Discussion

The bulletin boards are always placed in specific physical locations which afford a type of locally oriented text-, message- and meaning-making. In many cases the messages on the boards point to activities (e.g. upcoming religious meetings), events (a pet that has gone missing) or transactions, such as offers to buy a bicycle, that occur at walking distance from the bulletin board itself. From a semiotic perspective the bulletin board works as a medium for *indexical* signs (cf. [21,22]), pointing to everyday activities in the close vicinity of the board rather than to the global (or glocal) ongoings that are ever present in, for instance, social media. Perhaps, rather paradoxically, part of the explanation to why the bulletin board has survived in the digital era of the internet lies in its *immobility*. Because of the constant global access to and character of social media, but also due to the *mobility* of the devices through which they are accessed, texts and messages posted on, for instance Facebook, can never be as local as those posted on a physical bulletin board.

Rather than having replaced and made obsolete the analog bulletin board, emerging digital technologies have been integrated into the older medium through the still lively practice of printing and posting paper messages on surfaces designated for this type of communication. This is evidenced by the frequent use of intermedial links—telephone numbers, web pages, social media accounts, email addresses, QR-codes, etc.—that add a layer of affordances on top of the constraints that the bulletin board is limited by. The widespread diffusion of home printing technologies in Sweden has also expanded the possibilities of using clipart and digital images in color print, even for small business and private persons. Just as cheap consumer technologies combined with higher capacity infrastructure have changed how video and audio is distributed online on social media platforms such as Youtube and Vimeo, the bulletin board has been extended by other peripheral technologies.

In comparison to the results of Alt et al. [3] we argue that the “decline” in use of bulletin boards is still not resolved. We need historical data, which is representative (rather than based on convenience sampling), to draw such a conclusion. Our study, however, indicates an active use of the boards in the everyday communication of people and organizations, along the same lines as Churchill, Nelson and Denoue observed that “[i]nterviews with local residents indicated that poster boards serve an important communication function within communities” [5].

Moreover, Alt et al. propose a set of design principles for the future digital version of public notice areas. Our study shows that users are already connecting digital and analog media, but using them in slightly different ways. Messages that appear on the analog bulletin board are also simultaneously posted in online environments such as Facebook. Moreover, we show that intermediality is a common feature of analog messages, especially when linking to phone-numbers, hyperlinks and email addresses. This way, there already exists an ad-hoc design pattern created dynamically by the users themselves as the media landscape has changed. While many social media services have borrowed features from analog bulletin boards, we have shown that there also is a reverse movement of bulletin boards users adapting to new media.

With regards to what types of messages are represented on Swedish bulletin boards, our results show several similarities with the findings of Churchill, Nelson and Denoue. Their content analysis of the boards revealed that:

“[…] in the Mission District of San Francisco advertised dance and cooking classes, English lessons, yoga classes, religious gatherings and political meetings, while nearby Noe Valley poster boards sought and advertised baby sitters, dog walkers, hiking partners, lost pets, Pilates classes and, again, yoga classes” [5].

These results correspond well to what we found in messages posted by private persons and small companies. However, we found to a greater extent messages created by associations, such as sports clubs and churches. Even though Churchill, Nelson and Denoue do not quantify their results, the absence of associations and institutions as senders of messages in their material might indicate that there is a slightly different composition of the boards between the countries. In the study by Alt et al. [3], the institutional senders are better represented, even though it is difficult to compare our level of analysis (messages) with theirs (boards).

As the results of our analyses show, the bulletin board continues to fulfill an important function within the close and local surroundings of its placement contributing to maintenance of Swedish culture and social life. Typical senders of ‘local’ messages on the billboards are associations such as sports clubs, churches or local cultural groups. Such groups use the boards to spread invitations to local activities with the goal of gathering people in social activities and therefore also shaping Swedish cultural life. The study moreover shows how such senders and their invitations *mediate* [21] local places as sites for educational life with specific regard to spiritual, relational/emotional learning; embodied/physical learning; “Bildung”/ general education; school learning; and work-related/professional learning. These findings indicate how billboards mediate Swedish cultural life and function as what in educational terms could be referred to as a *curriculum of the local community*.

### Methodological limitations

In this study we often ran into classification issues, where it was difficult to make proper distinctions between messages. For example, drawing a boundary between a “private person” and a small business is sometimes hard to determine, since the legal definition is often not stated explicitly. This boundary is also difficult to draw between associations that are nonprofit or have a commercial purpose, and other forms of businesses. Furthermore, with the advances of printing technologies, there are instances of messages that can not be easily identified as either home- or professionally printed. Today, color laser printers are feasible investments both for small companies and private persons in Sweden. Moreover, we had to expand our codebook to include more and more media types that we did not expect to be present in the messages, such as QR-codes and social media user handles.

Another limitation concerns methodological representativity (as discussed above). Our results show a very strong domination of Swedish language messages (only 3% were in another language than Swedish, which can be compared to another study in Moldavia by Muth [23] which showed how public spaces preserved Russian language expressions despite the national policy stating that Romanian Moldovan is the official language). However, this is certainly not true for bulletin boards all across Sweden. In areas where there is a concentration of residents with native languages other than Swedish, the boards have a greater linguistic diversity in messages written by private persons, associations and companies. This is also true for locations that attract a large number of tourists, especially by small companies that offer services for visitors. For future studies that make use of the citizen science method for studying cultural phenomena, it is important to reflect on the composition of volunteer groups with regards to linguistic and cultural diversity, especially when the object of study is local to the volunteer communities. In particular, when engaging schools in citizen science driven data-gathering, inequalities in terms of economic and cultural resources are probably influencing which school classes participate (see also Svendsen [9] and Purschke [7,8]).

### Media trends and future work

We encourage communication scholars to conduct more studies on the intersections of digital and analog media and to make use of the open dataset that we have published here. One interesting phenomenon to investigate is the turnover of messages on bulletin boards. This would require longitudinal studies such as the one reported by Peck & Banda [6] where a selection of boards are followed over time. This way, it would be possible to assess how frequently the board is used, which messages are preserved or removed, to detect if there are moderation of censorship practices, and through in-depth qualitative analysis, find out more about the local needs and uses of this media technology. Furthermore, it would also be interesting to conduct interviews with the users of bulletin boards. This could be achieved by contacting the senders via electronic media and ask them why and how they use the bulletin board. Future studies of interest could also consist of comparative analysis between digital and analog media environments. Besides the example of a sports club using both analog messages and a Facebook group, we also identified a message about a missing pet that occurred both in our dataset and online in a local Facebook group for missing pets (this example was not discussed due to privacy concerns). This opens up for interesting comparative analysis between what Ivkovic and Lotherington call “Virtual Linguistic Landscapes” [22] and physical linguistic landscapes as represented in the bulletin board messages. While previous research often has analysed these two landscapes separately, our study indicates interesting connections between physical locations and intermedial connections to electronic media. As modern smartphones continue to be widely adopted and new location-based applications are becoming popular (for example Tinder, Facebook check-ins, Google Maps) we could draw many lessons from existing physical location-based media, both to understand media use and to develop new innovations for location-based electronic media.

## Supporting information

S1 Appendix Raw data from data collection. (CSV)

S2 Appendix Source code as a Jupyter/Python3 notebook. (IPYNB)

S3 Appendix Geodata for each bulletin board message. (CSV)

S4 Appendix Population demographics, Swedish cities, 2017. Provided by Statistics Sweden.
(XLSX)
